# Unusual Late Onset of Parenchymal Neuro-Behçet Disease

**DOI:** 10.1155/2016/5720319

**Published:** 2016-07-26

**Authors:** Wai Wai Miller, Demetrios Konstas, Chetan Gandhy, Derrick Robertson

**Affiliations:** ^1^Department of Neurology, University of South Florida, James A. Haley Veterans Hospital, 13000 Bruce B. Downs Boulevard VAH 127, Tampa, FL 33612, USA; ^2^Department of Neurology, University of South Florida, College of Medicine, Tampa, Fl 33612, USA

## Abstract

Neuro-Behçet disease (NBD) is a multisystem inflammatory disorder characterized by oral lesions, genital lesions, uveitis, and neurological deficits. If left untreated, it may lead to worsening neurological function and can be fatal. Here we present a case of a 52-year-old woman who was diagnosed with Behçet disease (BD) as a teenager and had a relatively mild disease course. Decades later after her initial DB diagnosis, she presented to our hospital with a chief complaint of headache. She did not have focal neurological deficits or any active mucosal lesions. Upon further investigation, the patient was found to have multiple inflammatory changes on neuroimaging and abnormal cerebrospinal fluid (CSF), consistent with the diagnosis of NBD. She was treated with intravenous corticosteroid therapy and her symptoms resolved. Although our patient presented with minimal symptoms decades after her initial diagnosis, any neurological complaint warranted a thorough investigation for a proper diagnosis and treatment given the multisystem involvement of BD.

## 1. Introduction

Behçet disease (BD) is a multisystem disorder that is characterized by oral-genital ulcers and uveitis. In Neuro-Behçet disease (NBD), the central nervous system involvement can be categorized as either parenchymal or nonparenchymal [1]. The typical age of onset is between 20 and 40 years for NBD, and men are more often affected than women [1]. In prior studies, the mean duration between the onset of NBD and time of developing BD ranged from 3 to 6 years [1]. In another study with a large cohort of NBD patients, the median duration from BD diagnosis until NBD was 6 years [2]. The diagnosis is made with clinical presentation, findings on magnetic resonance imaging (MRI), and cerebrospinal fluid (CSF) analysis. Here we present a patient who had an unusual late onset of NBD after she was diagnosed with BD decades earlier.

## 2. Case Presentation

Our patient is a 52-year-old Caucasian woman of German descent with a remote history of BD diagnosed as a teenager with recurrent oral lesions, genital lesions, and uveitis with only mild exacerbations over the past 30 years, who presented to us with a new onset headache. She reported that the headache started about 4 weeks before and it was intermittent at first but became constant 7 days prior to admission. She described the headache as pressure-like pain at the occipital regions bilaterally radiating to the vertex. Her headaches were associated with intermittent bilateral ear pain. She denied any focal weakness, nausea, vomiting, photophobia, or phonophobia. Aggravating factors for the headache included staring at the computer screen at work. She tried over-the-counter anti-inflammatories along with baclofen which helped initially. She denied any recent travel outside the US, had no recent sick contacts, reported infrequent alcohol use, and denied any toxic habits such as smoking and illicit drug use. She had no previous history of similar headaches personally or a family history of headaches. Physical examination, including a detailed neurologic examination, was within normal limits.

Of note, eight months prior to her presentation for evaluation of her headache, she had an episode of acute word-finding difficulties. She presented to our hospital and was admitted for a workup of suspected ischemic stroke. A brain MRI showed a focal T2 hyperintensity within the posterior left temporal lobe assumed to be due to an acute ischemic stroke. She was admitted and further workup was unrevealing of the etiology of her suspected ischemic stroke. As an outpatient, a repeat brain MRI one month after hospitalization showed resolution of this left temporal lesion. Clinically, the patient reported complete resolution of her word-finding difficulties.

**Figure 1:**
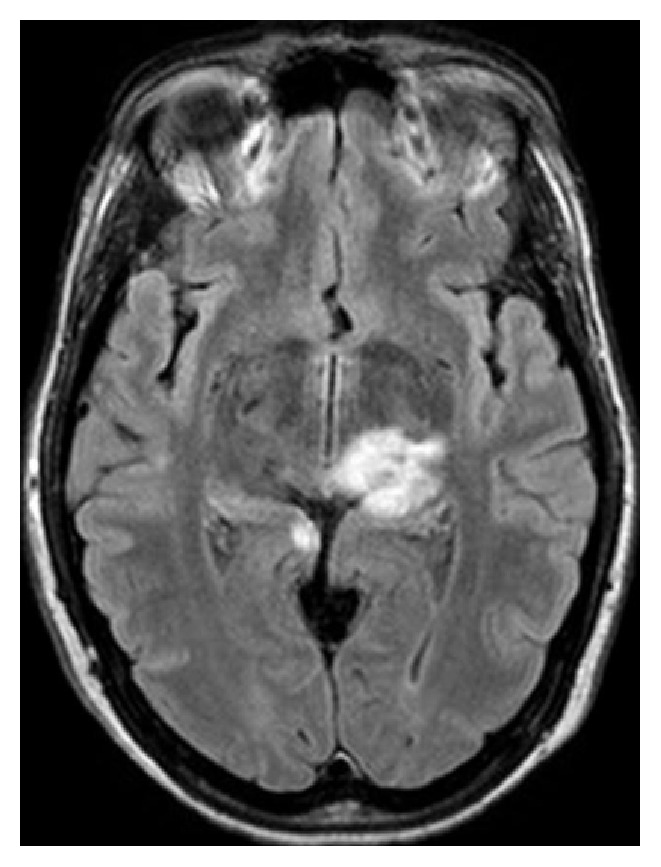
MRI brain T2 axial FLAIR sequence showed hyperintensities in the left thalamus and right mesial occipital lobe.

**Figure 2:**
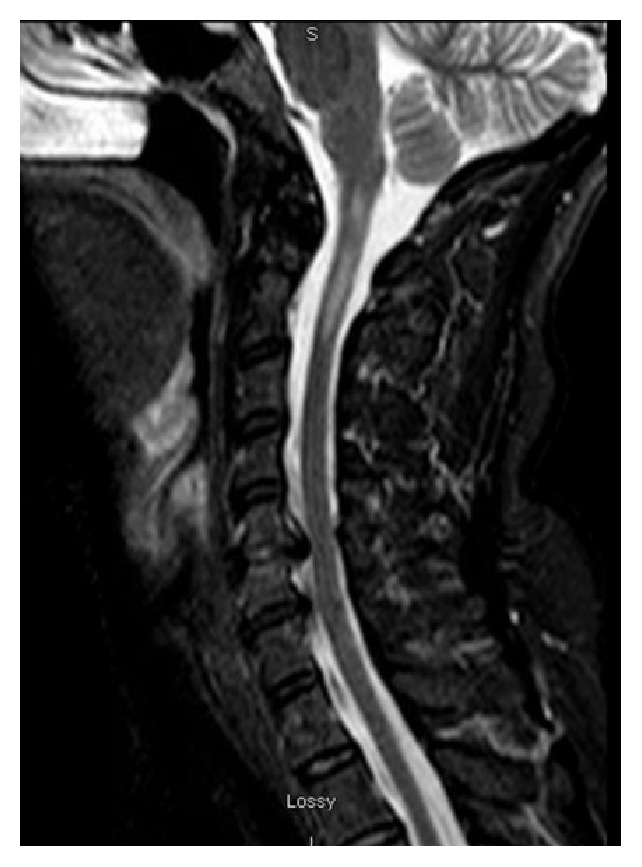
MRI cervical spine T2 sagittal sequence showed a hyperintensity extending from C1 to C2.

On the admission to evaluate her headache, a repeat brain MRI showed T2 hyperintensities in the left thalamus, right mesial occipital lobe, and left brachium pontis (Figure 1). The MRI of the cervical spine showed an abnormal T2 hyperintensity within the cervical spinal cord at the level of C1-2 (Figure 2). CSF analysis revealed elevated nucleated cells and protein at 114/*μ*L and 165 mg/dL, respectively. Additional CSF analyses, including glucose, angiotensin-converting enzyme levels, cytology, flow cytometry, IgG index, and oligoclonal banding, were all normal. She was treated with high-dose methylprednisolone leading to a complete resolution of her headache.

In regard to her past medical history of BD, she initially presented with recurrent oral ulcers and then a short time later developed recurrent genital ulcers and uveitis at age of 17. She was evaluated by multiple medical specialists and underwent an extensive workup. Subsequently, she was diagnosed with BD. She was started on oral prednisone, which she took for approximately 3 years and eventually self-discontinued because of prednisone side effects (weight gain, mood fluctuations). She had not had exacerbation with oral or eye lesions in over 30 years but did have infrequent bouts of genital ulcers. These exacerbations typically occurred in the setting of a stressor, such as an upper respiratory infection. Her last genital outbreak was one month prior to admission for headache. She had been tested before for HLA-B51, whose carriers have an increased risk of developing BD, but was negative and has no known family history of BD.

## 3. Discussion

NBD disease is classified as either parenchymal or nonparenchymal. Parenchymal disease accounts for 75% of cases, and headache is the most common neurological symptom, occurring in about 70% of patients [1]. NBD can be acute or chronic. Acute NBD is often seen as meningoencephalitis and typically responds well to corticosteroid therapy. Acute NBD tends to be self-limiting and carries a better prognosis as opposed to chronic progressive NBD, which is intractable and often times leads to severe disability and deterioration [3]. Some patients may not present with any overt neurological deficits but rather with vague nonfocal neurological symptoms, such as headache or dizziness [4]. Our patient presented to the hospital with new onset headache being the only symptom and no other neurological deficits were noted. She received a high-dose intravenous corticosteroid treatment and her symptoms quickly resolved. This subclinical acute NBD may be mistaken for chronic progressive NBD; however they have different prognoses. Although the etiology is still unclear, this subclinical NBD is potentially thought to be a different form of NBD rather than the earlier stages of chronic progressive NBD [4]. Further studies are needed to better define this.

Just like clinical symptoms, MRI findings are also nonspecific in NBD. Typically, there is a single lesion during the acute phase, but the lesions can be diffuse and widespread during the chronic phase [1]. The shape of the lesions seen on T2-weighted images varies from circular, linear, or crescent-shaped to irregular [5]. Bilateral lesions are uncommon [1]. Lesions are typically located in the brain stem, thalamus, or basal ganglia and decrease in size with steroid or other immunosuppressive therapies [5]. The presence of brainstem atrophy can be seen in chronic progressive NBD [6].

CSF analysis in NBD typically reveals increased cell counts and protein. CSF neutrophilia can be seen in the early stages with lymphocytosis being seen in the later stage [1]. Oligoclonal bands are usually absent. Our patient's CSF analysis was consistent with NBD as it showed elevated cell counts with neutrophilia, elevated protein, and absent oligoclonal bands.

Estimation of prognosis for NBD is based on many different factors. In patients with chronic progressive NBD, the presence of brainstem atrophy and abnormal CSF findings have a poorer prognosis [1]. Also, patients with spinal cord and brainstem lesions are more likely to have incomplete recovery and thus have higher levels of disability [1]. Our patient's presentation is consistent with the most common type, parenchymal NBD. She did not have brainstem atrophy seen on neuroimaging but had lesions in cervical spinal cord and abnormal CSF findings, thus making an accurate determination of her prognosis difficult. Additionally, one-third of the patients with parenchymal NBD may relapse or become progressive even after appropriate treatment [7]. Therefore, our patient received prolonged corticosteroid taper upon discharge from the hospital and potentially will require long-term immunosuppressive agents.

Here we presented a patient with a myriad of uncommon NBD findings. She was a female over the age of 50 years who has had a relatively benign disease course of her BD for several decades. As mentioned above, NBD typically is diagnosed in the 3rd or 4th decade of life, presentation is 3 to 6 years after the onset of BD and it occurs more often in men. She did not present with systemic mucosal lesions or uveitis at the time of admission, contrary to the typical presentation of parenchymal NBD which often has concurrent systemic features of BD [1]. Some of the locations of the lesions on her MRI were uncommon in that they occurred bilaterally and affected the cervical spinal cord. Further confounding her clinical picture was her initial admission, months prior to the diagnosis of NBD, with acute word-finding difficulties. It is possible that this was the beginning of NBD in our patient; however the lack of a full diagnostic workup, including CSF analysis, makes that determination impossible retrospectively.

In conclusion, the diagnosis of NBD should be made cautiously in patients above the age of 50 years. Specifically excluding other more common neurological disorders, particularly stroke, is critical in obtaining a proper diagnosis. NBD should be considered in the differential diagnosis of any BD patient with new headache or other neurological complaints regardless of the prior status of their BD including disease duration and level of disability.
